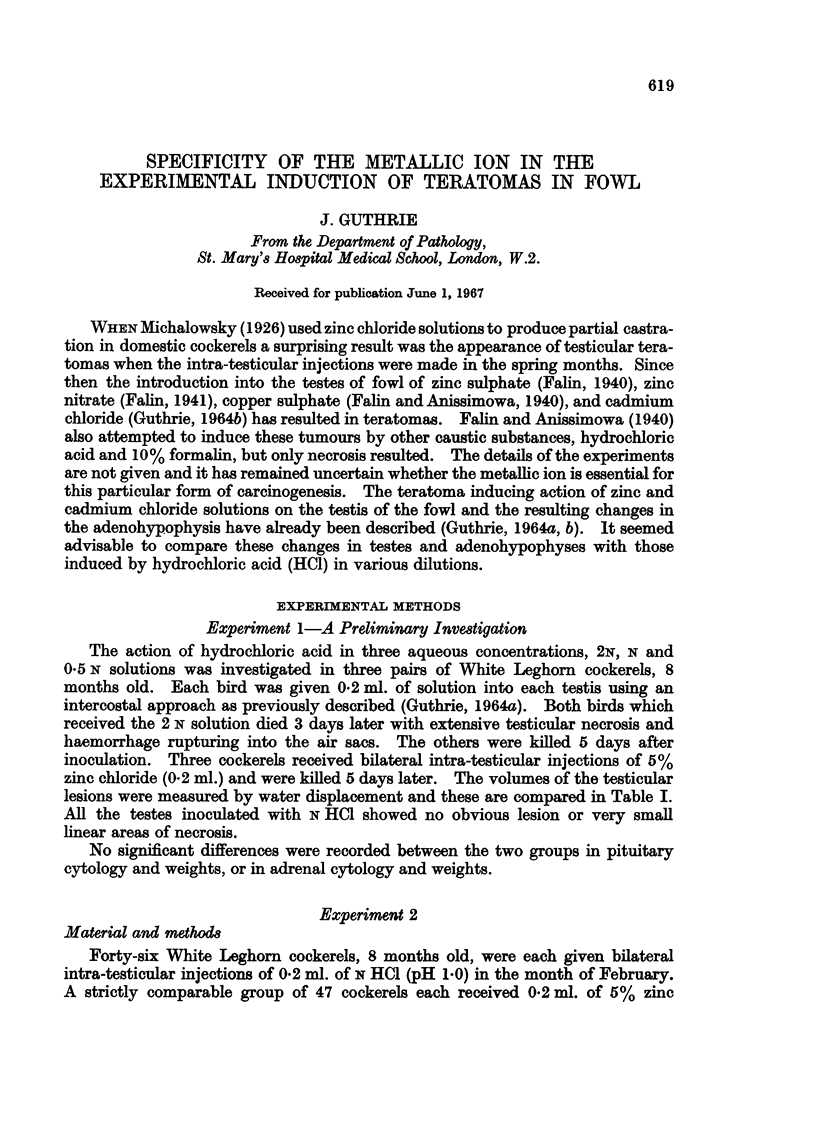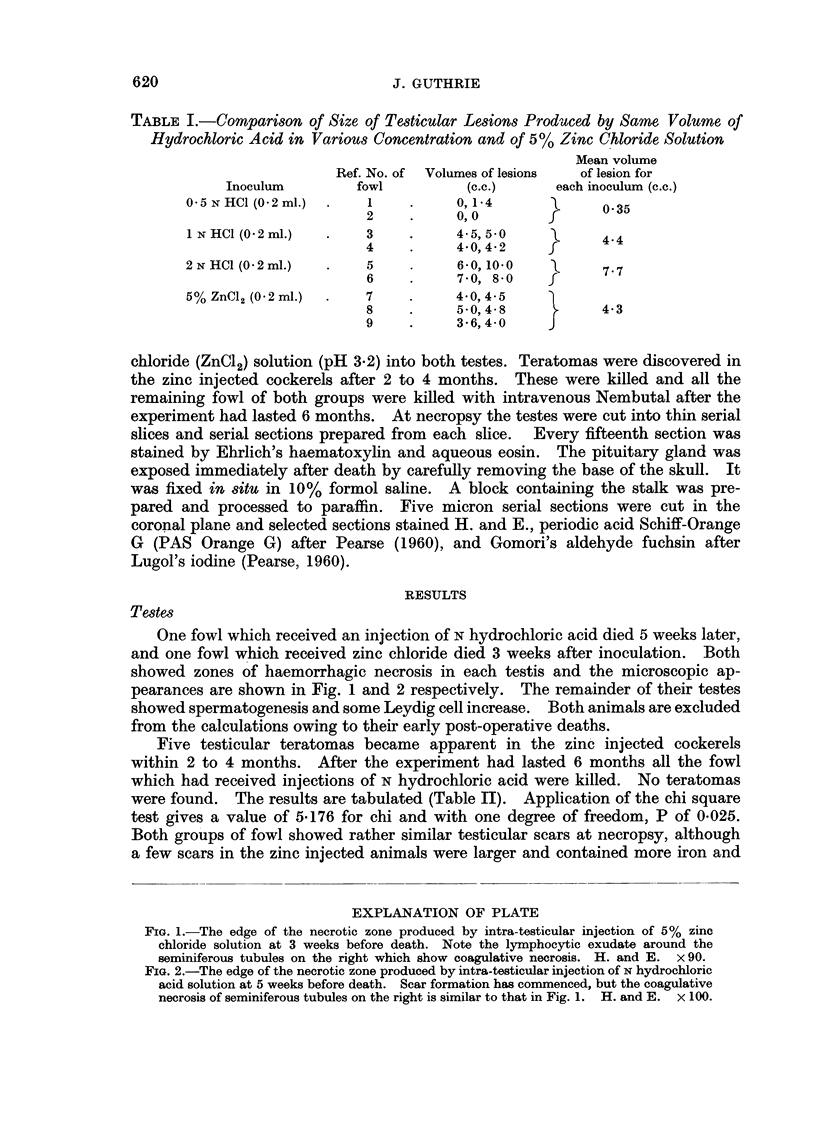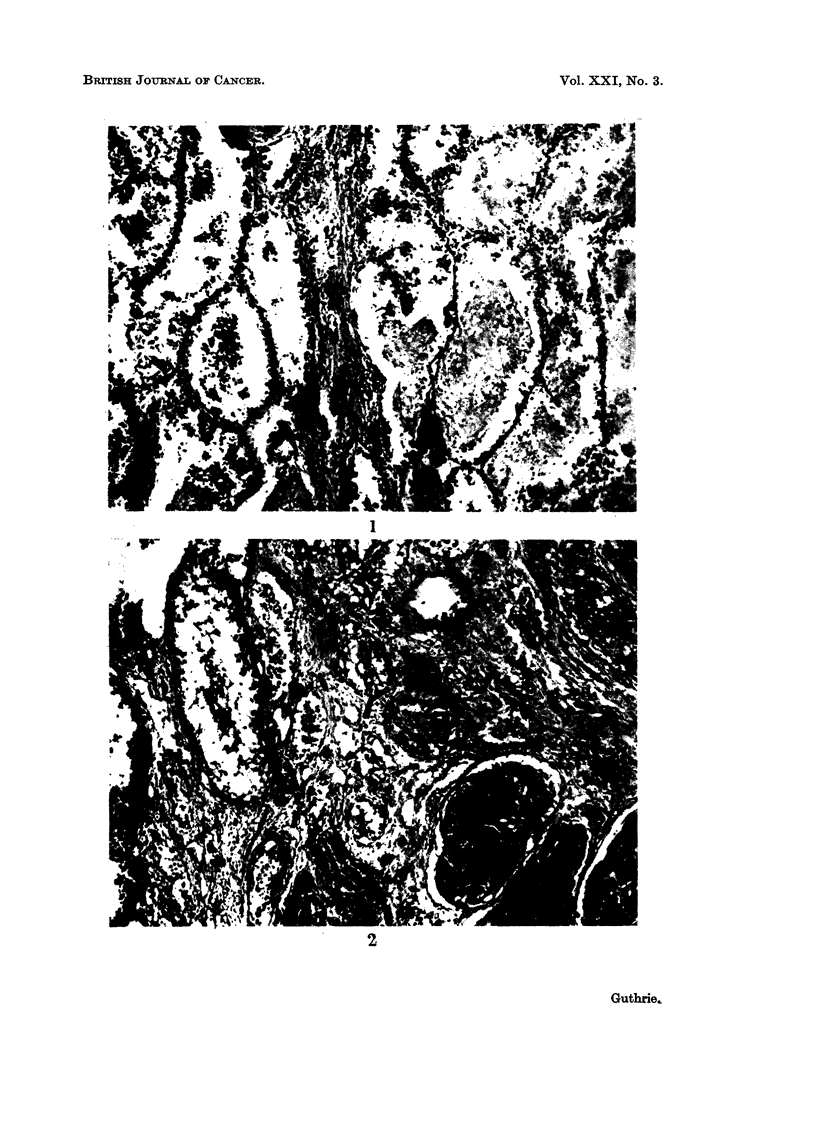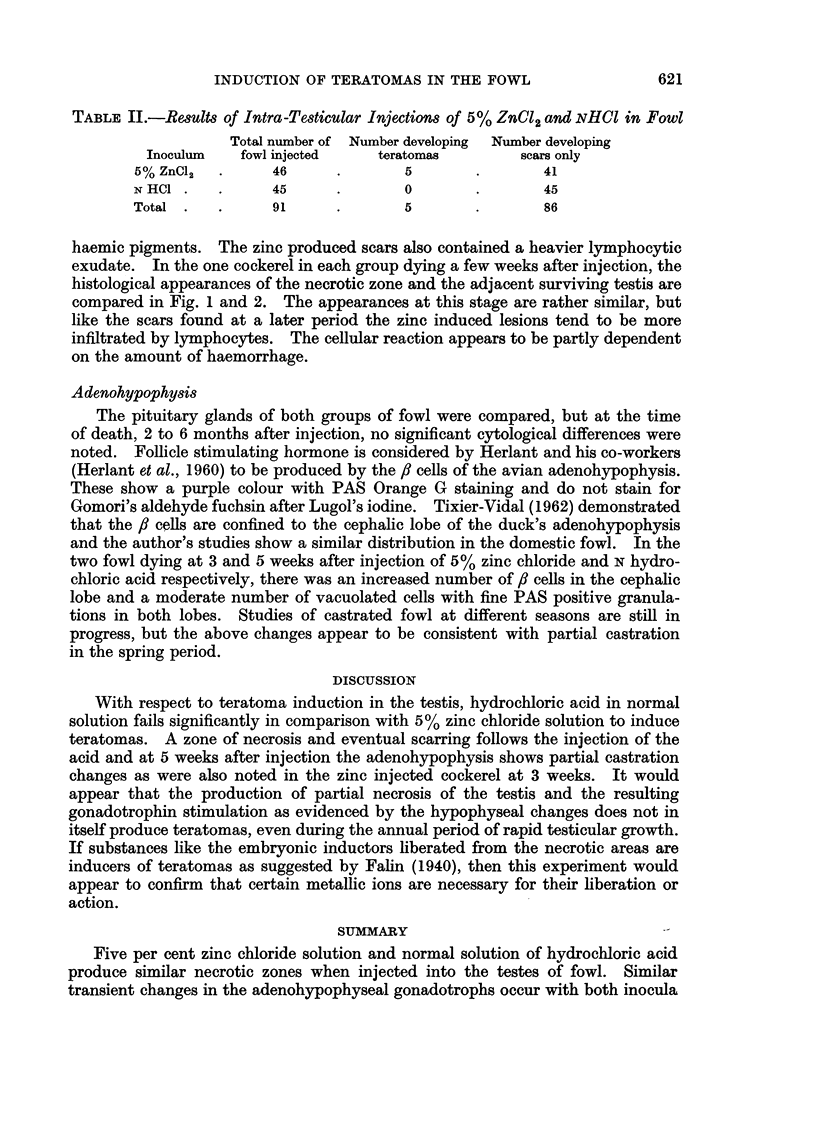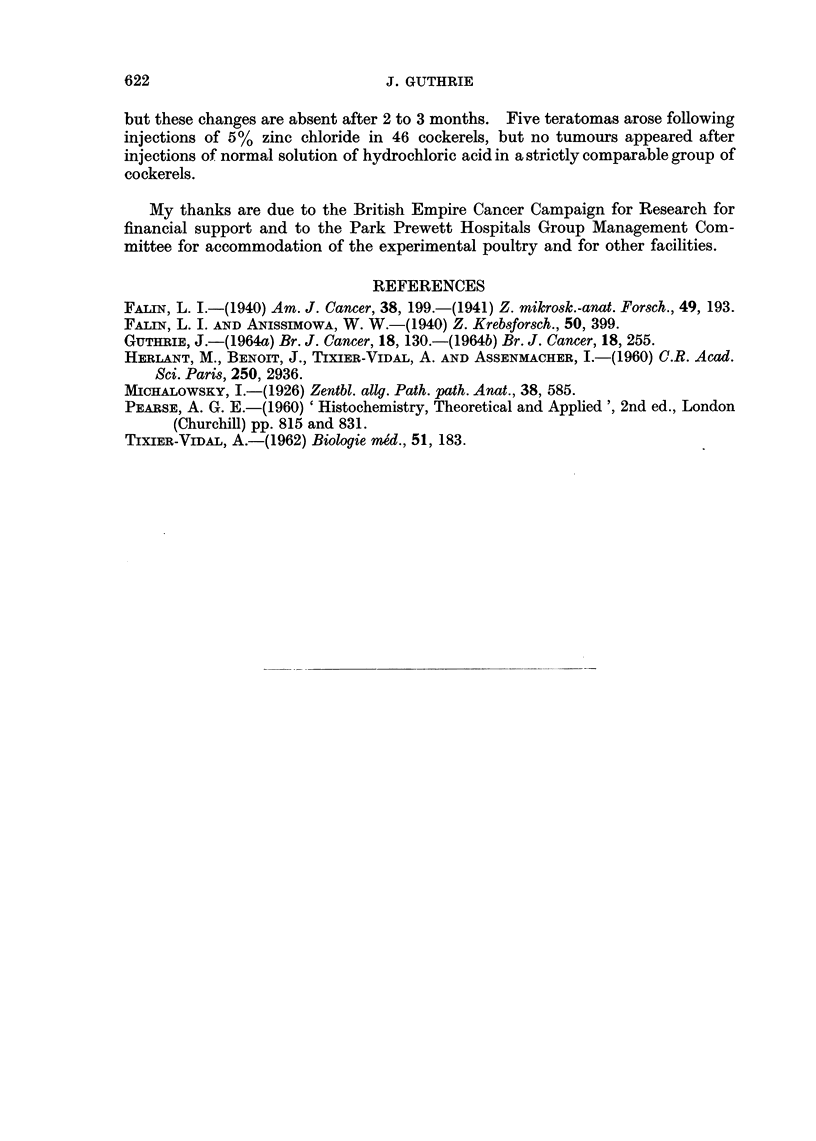# Specificity of the metallic ion in the experimental induction of teratomas in fowl.

**DOI:** 10.1038/bjc.1967.72

**Published:** 1967-09

**Authors:** J. Guthrie

## Abstract

**Images:**


### SPECIFICITY OF THE METALLIC ION IN THE EXPERIMENTAL INDUCTION OF TERATOMAS IN FOWL

J. GUTHRIE
*From the Department of Pathology,*
*St. Mary's Hospital Medical School, London, W.2.*

Received for publication June 1, 1967

WHEN Michalowsky (1926) used zinc chloride solutions to produce partial castration in domestic cockerels a surprising result was the appearance of testicular teratomas when the intra-testicular injections were made in the spring months. Since then the introduction into the testes of fowl of zinc sulphate (Falin, 1940), zinc nitrate (Falin, 1941), copper sulphate (Falin and Anissimowa, 1940), and cadmium chloride (Guthrie, 1964*b*) has resulted in teratomas. Falin and Anissimowa (1940) also attempted to induce these tumours by other caustic substances, hydrochloric acid and 10% formalin, but only necrosis resulted. The details of the experiments are not given and it has remained uncertain whether the metallic ion is essential for this particular form of carcinogenesis. The teratoma inducing action of zinc and cadmium chloride solutions on the testis of the fowl and the resulting changes in the adenohypophysis have already been described (Guthrie, 1964*a*, *b*). It seemed advisable to compare these changes in testes and adenohypophyses with those induced by hydrochloric acid (HCl) in various dilutions.

### EXPERIMENTAL METHODS

### *Experiment* 1—*A Preliminary Investigation*

The action of hydrochloric acid in three aqueous concentrations, 2N, N and 0·5 N solutions was investigated in three pairs of White Leghorn cockerels, 8 months old. Each bird was given 0·2 ml. of solution into each testis using an intercostal approach as previously described (Guthrie, 1964*a*). Both birds which received the 2 N solution died 3 days later with extensive testicular necrosis and haemorrhage rupturing into the air sacs. The others were killed 5 days after inoculation. Three cockerels received bilateral intra-testicular injections of 5% zinc chloride (0·2 ml.) and were killed 5 days later. The volumes of the testicular lesions were measured by water displacement and these are compared in Table I. All the testes inoculated with N HCl showed no obvious lesion or very small linear areas of necrosis.

No significant differences were recorded between the two groups in pituitary cytology and weights, or in adrenal cytology and weights.

### *Experiment* 2

*Material and methods*

Forty-six White Leghorn cockerels, 8 months old, were each given bilateral intra-testicular injections of 0·2 ml. of N HCl (pH 1·0) in the month of February. A strictly comparable group of 47 cockerels each received 0·2 ml. of 5% zinc

TABLE I.—*Comparison of Size of Testicular Lesions Produced by Same Volume of Hydrochloric Acid in Various Concentration and of 5% Zinc Chloride Solution*

| Inoculum | Ref. No. of fowl | Volumes of lesions (c.c.) | Mean volume of lesion for each inoculum (c.c.) |
|---|---|---|---|
| 0·5 N HCl (0·2 ml.) | 1 | 0, 1·4 | 0·35 |
| | 2 | 0, 0 | |
| 1 N HCl (0·2 ml.) | 3 | 4·5, 5·0 | 4·4 |
| | 4 | 4·0, 4·2 | |
| 2 N HCl (0·2 ml.) | 5 | 6·0, 10·0 | 7·7 |
| | 6 | 7·0, 8·0 | |
| 5% $ZnCl_2$ (0·2 ml.) | 7 | 4·0, 4·5 | 4·3 |
| | 8 | 5·0, 4·8 | |
| | 9 | 3·6, 4·0 | |

chloride ($ZnCl_2$) solution (pH 3·2) into both testes. Teratomas were discovered in the zinc injected cockerels after 2 to 4 months. These were killed and all the remaining fowl of both groups were killed with intravenous Nembutal after the experiment had lasted 6 months. At necropsy the testes were cut into thin serial slices and serial sections prepared from each slice. Every fifteenth section was stained by Ehrlich's haematoxylin and aqueous eosin. The pituitary gland was exposed immediately after death by carefully removing the base of the skull. It was fixed *in situ* in 10% formol saline. A block containing the stalk was prepared and processed to paraffin. Five micron serial sections were cut in the coronal plane and selected sections stained H. and E., periodic acid Schiff-Orange G (PAS Orange G) after Pearse (1960), and Gomori's aldehyde fuchsin after Lugol's iodine (Pearse, 1960).

### RESULTS

### *Testes*

One fowl which received an injection of N hydrochloric acid died 5 weeks later, and one fowl which received zinc chloride died 3 weeks after inoculation. Both showed zones of haemorrhagic necrosis in each testis and the microscopic appearances are shown in Fig. 1 and 2 respectively. The remainder of their testes showed spermatogenesis and some Leydig cell increase. Both animals are excluded from the calculations owing to their early post-operative deaths.

Five testicular teratomas became apparent in the zinc injected cockerels within 2 to 4 months. After the experiment had lasted 6 months all the fowl which had received injections of N hydrochloric acid were killed. No teratomas were found. The results are tabulated (Table II). Application of the chi square test gives a value of 5·176 for chi and with one degree of freedom, P of 0·025. Both groups of fowl showed rather similar testicular scars at necropsy, although a few scars in the zinc injected animals were larger and contained more iron and

---

### EXPLANATION OF PLATE

FIG. 1.—The edge of the necrotic zone produced by intra-testicular injection of 5% zinc chloride solution at 3 weeks before death. Note the lymphocytic exudate around the seminiferous tubules on the right which show coagulative necrosis. H. and E. ×90.

FIG. 2.—The edge of the necrotic zone produced by intra-testicular injection of N hydrochloric acid solution at 5 weeks before death. Scar formation has commenced, but the coagulative necrosis of seminiferous tubules on the right is similar to that in Fig. 1. H. and E. ×100.

1

2

Guthrie.

TABLE II.—*Results of Intra-Testicular Injections of* 5% $ZnCl_2$ *and* N*HCl in Fowl*

| Inoculum | Total number of fowl injected | Number developing teratomas | Number developing scars only |
|---|---|---|---|
| 5% $ZnCl_2$ | 46 | 5 | 41 |
| N HCl | 45 | 0 | 45 |
| Total | 91 | 5 | 86 |

haemic pigments. The zinc produced scars also contained a heavier lymphocytic exudate. In the one cockerel in each group dying a few weeks after injection, the histological appearances of the necrotic zone and the adjacent surviving testis are compared in Fig. 1 and 2. The appearances at this stage are rather similar, but like the scars found at a later period the zinc induced lesions tend to be more infiltrated by lymphocytes. The cellular reaction appears to be partly dependent on the amount of haemorrhage.

*Adenohypophysis*

The pituitary glands of both groups of fowl were compared, but at the time of death, 2 to 6 months after injection, no significant cytological differences were noted. Follicle stimulating hormone is considered by Herlant and his co-workers (Herlant *et al.*, 1960) to be produced by the $\beta$ cells of the avian adenohypophysis. These show a purple colour with PAS Orange G staining and do not stain for Gomori's aldehyde fuchsin after Lugol's iodine. Tixier-Vidal (1962) demonstrated that the $\beta$ cells are confined to the cephalic lobe of the duck's adenohypophysis and the author's studies show a similar distribution in the domestic fowl. In the two fowl dying at 3 and 5 weeks after injection of 5% zinc chloride and N hydrochloric acid respectively, there was an increased number of $\beta$ cells in the cephalic lobe and a moderate number of vacuolated cells with fine PAS positive granulations in both lobes. Studies of castrated fowl at different seasons are still in progress, but the above changes appear to be consistent with partial castration in the spring period.

### DISCUSSION

With respect to teratoma induction in the testis, hydrochloric acid in normal solution fails significantly in comparison with 5% zinc chloride solution to induce teratomas. A zone of necrosis and eventual scarring follows the injection of the acid and at 5 weeks after injection the adenohypophysis shows partial castration changes as were also noted in the zinc injected cockerel at 3 weeks. It would appear that the production of partial necrosis of the testis and the resulting gonadotrophin stimulation as evidenced by the hypophyseal changes does not in itself produce teratomas, even during the annual period of rapid testicular growth. If substances like the embryonic inductors liberated from the necrotic areas are inducers of teratomas as suggested by Falin (1940), then this experiment would appear to confirm that certain metallic ions are necessary for their liberation or action.

### SUMMARY

Five per cent zinc chloride solution and normal solution of hydrochloric acid produce similar necrotic zones when injected into the testes of fowl. Similar transient changes in the adenohypophyseal gonadotrophs occur with both inocula but these changes are absent after 2 to 3 months. Five teratomas arose following injections of 5% zinc chloride in 46 cockerels, but no tumours appeared after injections of normal solution of hydrochloric acid in a strictly comparable group of cockerels.

My thanks are due to the British Empire Cancer Campaign for Research for financial support and to the Park Prewett Hospitals Group Management Committee for accommodation of the experimental poultry and for other facilities.

### REFERENCES

FALIN, L. I.—(1940) *Am. J. Cancer*, **38**, 199.—(1941) *Z. mikrosk.-anat. Forsch.*, **49**, 193.
FALIN, L. I. AND ANISSIMOWA, W. W.—(1940) *Z. Krebsforsch.*, **50**, 399.
GUTHRIE, J.—(1964*a*) *Br. J. Cancer*, **18**, 130.—(1964*b*) *Br. J. Cancer*, **18**, 255.
HERLANT, M., BENOIT, J., TIXIER-VIDAL, A. AND ASSENMACHER, I.—(1960) *C.R. Acad. Sci. Paris*, **250**, 2936.
MICHALOWSKY, I.—(1926) *Zentbl. allg. Path. path. Anat.*, **38**, 585.
PEARSE, A. G. E.—(1960) ' Histochemistry, Theoretical and Applied ', 2nd ed., London (Churchill) pp. 815 and 831.
TIXIER-VIDAL, A.—(1962) *Biologie méd.*, **51**, 183.